# The ‘GA²LEN Sinusitis Cohort’: an introduction

**DOI:** 10.1186/2045-7022-5-S4-O1

**Published:** 2015-06-26

**Authors:** Griet Vandeplas, Asif Khan, Thi Minh Thao Huynh, Peter Tomassen, Thibaut van Zele, Lars-Olaf Cardell, Julia Arebro, Heidi Olze, Ulrike Foerster, Marek Leszek Kowalski, Agnieszka Olszewska-Ziąber, Wytske Fokkens, Cornelis van Drunen, Joaquim Mullol, Peter Hellings, Valérie Hox, Elina Toskala, Glenis Scadding, Valerie Lund, Claus Bachert

**Affiliations:** 1 Ghent University, Upper Airways Research Laboratory, Ghent, Belgium; 2 Sanofi, Health Economics and Outcomes Research, Paris, France; 3 AIXIAL, Biometry Department, Levallois-Perret, France; 4 Karolinska Institutet, Division of ENT Diseases, CLINTEC, Stockholm, Sweden; 5 Charité-Universitätsmedizin Berlin, Dept of Otorhinolaryngology, Berlin, Germany; 6 Medical University of Lodz, Dept of Immunology, Rheumatology and Allergy, Lodz, Poland; 7 Academic Medical Centre, Dept of Otorhinolaryngology, Amsterdam, Netherlands; 8 IDIBAPS, Clinical & Experimental Respiratory Immunoallergy, Barcelona, Catalonia, Spain; 9 University Hospitals Leuven, Dept of Otorhinolaryngology, Head and Neck Surgery, Leuven, Belgium; 10 Temple University School of Medicine, Dept of Otolaryngology-Head and Neck Surgery, Philadelphia, PA, USA; 11 Royal National Throat, Nose and Ear Hospital, Allergy and Medical Rhinology Unit, London, UK; 12 Royal National Throat, Nose and Ear Hospital, Rhinology Unit, London, UK

## Background

The Global Allergy and Asthma European Network (GA^2^LEN) is a network of the leading European allergy clinical and research facilities and the GA^2^LEN Sinusitis Cohort is a database within this network. The aim of this cohort is to intensify research on rhinosinusitis pheno- and endotypes, thus differentiating chronic rhinosinusitis (CRS) into smaller disease entities based on clinical, biological, and patient-reported outcomes.

## Methods

Patients (N = 869) from 9 participating outpatient ENT clinics recruited to participate in this cross-sectional, multicentre cohort study were assigned to 3 groups: CRS with nasal polyps (CRSwNP) and CRS without nasal polyps (CRSsNP), both diagnosed using EPOS 2012 guidelines; and control defined as an unmatched cohort of patients undergoing nasal turbinate surgery with or without allergy symptoms. Patient subgroups were compared by clinician reported, patient reported, imaging, endoscopy, lung function, and biomarker outcomes.

## Results

Overall, 51.2%, 27.3%, and 21.5% of patients were in the CRSwNP, CRSsNP, and control groups, respectively. Across groups, mean age was 42.1 years (range 15–76 years), 55.0% were male, and 95.5% were Caucasian. Comorbid allergic rhinitis was diagnosed in 33.2%, 29.0%, and 23.8% of patients in the CRSwNP, CRSsNP, and control groups, respectively. Of all patients, 33.2% had comorbid asthma, which was significantly (p < 0.0001) more frequent in the CRSwNP (49.6%) than CRSsNP (20.2%) group. CRSwNP patients reported late age of onset for asthma, whereas CRSsNP patients reported early age of onset. Among CRSwNP and CRSsNP patients who did not report asthma, 59.8% and 41.9%, respectively, had an FEV_1_/FVC ratio of ≤ 80% (p = 0.063). Contact allergy was present significantly more often in CRSsNP than CRSwNP patients (15.1% vs 5.2%; p = 0.0001). Overall, 4.4% of all patients reported aspirin intolerance, which was significantly more frequent in CRSwNP vs CRSsNP patients (7.4% vs 1.3%; p < 0.0001); among these CRSwNP patients, 90.9% had asthma, forming the Aspirin Exacerbated Respiratory Disease (AERD) subgroup. A significantly higher number of CRSwNP patients reported previous surgery vs CRSsNP patients (45.9% vs 22.7%; p < 0.0001).

## Discussion

The GA^2^LEN Sinusitis cohort was not intended to be population representative; however, it is a large cohort of patients recruited throughout Europe with clinical, biomarker, as well as patient-reported outcomes.

